# *ADGRL3* genomic variation implicated in neurogenesis and ADHD links functional effects to the incretin polypeptide GIP

**DOI:** 10.1038/s41598-022-20343-z

**Published:** 2022-09-23

**Authors:** Oscar M. Vidal, Jorge I. Vélez, Mauricio Arcos-Burgos

**Affiliations:** 1 Department of Medicine, Universidad del Norte, Km 5 vía Puerto Colombia, 081007 Barranquilla, Colombia; 2 Grupo de Investigación en Psiquiatría (GIPSI), Departamento de Psiquiatría, Facultad de Medicina, Instituto de Investigaciones Médicas, Universidad de Antioquia, 050010 Medellín, Colombia; 3 Department of Industrial Engineering, Universidad del Norte, Km 5 vía Puerto Colombia, 081007 Barranquilla, Colombia

**Keywords:** Behavioural genetics, Genetic markers, ADHD, Computational models, Protein structure predictions

## Abstract

Attention deficit/hyperactivity disorder (ADHD) is the most common childhood neurodevelopmental disorder. Single nucleotide polymorphisms (SNPs) in the Adhesion G Protein-Coupled Receptor L3 (*ADGRL3*) gene are associated with increased susceptibility to developing ADHD worldwide. However, the effect of *ADGRL3* non-synonymous SNPs (nsSNPs) on the ADGRL3 protein function is vastly unknown. Using several bioinformatics tools to evaluate the impact of mutations, we found that nsSNPs rs35106420, rs61747658, and rs734644, previously reported to be associated and in linkage with ADHD in disparate populations from the world over, are predicted as pathogenic variants. Docking analysis of rs35106420, harbored in the *ADGLR3-*hormone receptor domain (HRM, a common extracellular domain of the secretin-like GPCRs family), showed that HRM interacts with the Glucose-dependent insulinotropic polypeptide (GIP), part of the incretin hormones family. GIP has been linked to the pathogenesis of diabetes mellitus, and our analyses suggest a potential link to ADHD. Overall, the comprehensive application of bioinformatics tools showed that functional mutations in the *ADGLR3* gene disrupt the standard and wild ADGRL3 structure, most likely affecting its metabolic regulation. Further in vitro experiments are granted to evaluate these in silico predictions of the ADGRL3-GIP interaction and dissect the complexity underlying the development of ADHD.

## Introduction

Attention deficit/hyperactivity disorder (ADHD) is a harmonic temporal-spatial neurodevelopment disorder characterized by persistent and age-inappropriate patterns of inattention, hyperactivity, and impulsivity^1–5^. Neurodevelopment is orchestrated by cellular and molecular-guided events in which cell communication is delivered through a series of molecular receptors and ligands that allow cells to switch between proliferation, migration, and differentiation^6,7^. Hence, molecular dysregulation may disturb the balance in a series of well-orchestrated cellular cascades related to the development of ADHD, *i.e.,* neurite outgrowth and axon guidance^8^.

Although ADHD aetiology is multifactorial^9^, genetic factors play a significant role. Estimates suggest that the heritability using family-based studies is 74%^10^, which is a figure significantly higher than that of up to 21.6% estimated by genome-wide association studies (GWASs)^3,11^. Thus, much more research must be done to elucidate the fundamental cause of this “missing heritability”.

Adhesion G protein-coupled receptors (aGPCRs) represent the five prominent families in the GPCR signaling pathway superfamily. The GPCRs are molecularly responsive to extracellular ligands such as chemical hormones, neurotransmitters, and peptides^12^. They exhibit a large multi-domain N-termini and a highly conserved region, prone to be cleaved by auto proteolysis^13^. GPCRs also influence many critical neurophysiological and neurodevelopmental processes and cellular responses, which are still not completely understood^14^.

A subfamily of aGPCR, named latrophilins, plays a significant role in neurophysiological processes^15^. One of the latrophilins members, *ADGRL3* (formerly known as *LPHN3*; latrophilin 3), has been linked to signal cell–cell adhesion, neuron guidance, and signal transduction^2,16^ and plays an essential role in the development of the central nervous system (CNS) during childhood^17^. ADGRL3, the protein encoded by the *ADGRL3* gene, along with ADGRL1 (latrophilin 1), ADGRL2 (latrophilin 2), and ADGRL4 (ELTD1)^18,19^, have orthologs in vertebrate and invertebrate species^20^.

**Figure 1:**
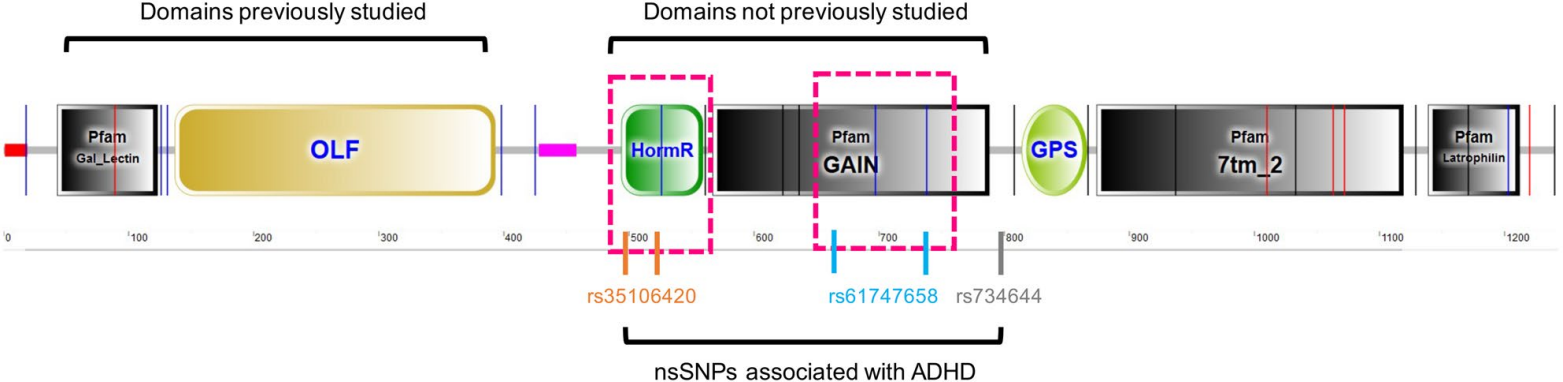
Schematic representation of ADGRL3 and its domains. Squares in pink represent the encoding genome areas of the protein harboring functional nsSNPs as defined by the in-silico analysis. Gal_Lectin: Galactose binding lectin domain (PF02140.18); OLF: Olfactomedin-like domain (PF02191.16); HRM: Hormone receptor domain (PF02793.22); GAIN: GPCR-autoproteolysis inducing domain (PF16489.5); GPS: GPCR proteolysis site motif (PF01825.21); 7tm_2: 7 transmembrane receptors (Secretin family; PF00002.24); Latrophilin: Latrophilin Cytoplasmic C-terminal region (PF02354.16). *ADGRL3* nsSNPs studied herein are located within the region of the HRM and GAIN domains.

Latrophilins are structurally composed of two adhesion protein domains, lectin, and olfactomedin, followed by a hormone-binding domain next to a GPCR autoproteolytic inducing (GAIN) domain (Fig. 1). This domain contains a post-translational modification cleavage process at the GPCR proteolysis site (GPS) motif, part of the larger GPCR-Autoproteolysis INducing (GAIN) domain conserved seven-transmembrane domain, and a C-terminal tail (Fig. 1). This autoproteolysis domain generates two sub-molecules: an extracellular N-terminal fragment (NTF) and a C-terminal fragment (CTF). After catalysis, both fragments are still non-covalently linked to the cell membrane^18,21^.

ADGRL3 modulates neuronal interaction and axon guidance and synaptic plasticity, all processes highly controlled and orchestrated during CNS development^17,22^. Furthermore, genetic variants in the *ADGRL3* gene have been associated with ADHD susceptibility through a series of population genetic analyses in distinct regions and ethnicities^23–31^, making *ADGRL3* one of the genes prominently associated with ADHD^3,32–34^. *ADGRL3* variants predispose to ADHD, modulate brain metabolism, and predict ADHD severity, ADHD comorbidity with conduct disorder (CD), oppositional defiant disorder (ODD), substance use disorder (SUD), and response to stimulant treatment^3,4,23,24,26,31,34–37^. However, it is still unclear how non-synonymous SPNs (nsSNPs) may affect the role of *ADGRL3* in ADHD pathogenesis. To the best of our knowledge, the potential pathogenicity of *ADGRL3* nsSNPs has not yet been studied.

Through structural modeling, in-silico approaches have been applied to identify the effect of deleterious nsSNPs in candidate genes^38,39^. This study combined a series of computational models to understand the pathogenic potential of different *ADGRL3* nsSNPs previously reported to confer susceptibility to ADHD^23,26,30,35,40^. We hypothesize that *ADGRL3* nsSNPs potentially dysregulate ADGRL3 stability and function^41,42^ and affect amino acid conservation, protein stability, structural composition, and protein–protein interactions.

Here, we showed a potential interaction between a specific region of ADGRL3, the hormone receptor (HRM) domain, and the Glucose-dependent insulinotropic polypeptide/Gastric inhibitory polypeptide (GIP) of the secretin family. GIP polypeptide has been shown to play a critical role in neuronal biogenesis and neural cell division. This significant event could modulate neuroplasticity during embryogenesis, which is one of the critical etiological factors related to ADHD. Furthermore, GIP promotes cell proliferation and is expressed in many organs and tissues, including the central nervous system^7^. Therefore, we hypothesize that GIP and its role in glucose metabolism could be a pathogenic factor linking ADHD and metabolic disorders such as diabetes.

## Results

### Collection and collation of ADGRL3 nsSNP data

We retrieved 1013 nsSNPs related to the *ADGRL3* gene from three databases (977 from the NCBI, 12 from the DisGeNET, and 24 from nsSNPdpe) (Table 1S, Supplementary Material) and nsSNPs from case/control- and family-based genetic studies on ADHD. After manually screening and eliminating duplicate records, we found three nsSNPs reported at least in two databases (Table 1).

**Table 1:** *ADGRL3* nsSNPs as reported in different databases and ADHD association studies.

nsSNP	NCBI	DisGeNET	nsSNPdpe	Reported in ADHD association studies	References
rs35106420	Yes	No	Yes	Yes	^23,35^
rs61747658	Yes	No	Yes	Yes	^35,40^
rs734644^a^	Yes	Yes	No	Yes	^30^

*SNP* single nucleotide polymorphism.

^a^According to HaploReg v 4.1^110^, only this marker is synonymous.

### Prediction of ADGRL3 nsSNPs pathogenicity

Several pathogenicity prediction software tools were used to predict the pathogenicity of three selected *ADGRL3* nsSNPs (Table 2a), including Mutpred2, PANTHER-PSEP, PhD-SNP, PolyPhen 2.0, PROVEAN, and SIFT. Pathogenic mutations were not predicted by MutPred2 nor PhD-SNP. However, PANTHER predicted R465Q (rs35106420) and R533Q (rs35106420) as “probably damaging” (time > 455my), and T659A, T727A, and N693K (rs734644) as “possibly damaging” (> 200my) (Table 2a). Similarly, PolyPhen 2.0 predicted R465Q (rs35106420), R533Q (rs35106420), and N693K (rs734644) to be pathogenic. On the other hand, PROVEAN only considered R533Q (rs35106420) to be pathogenic, while SIFT considered R465Q (rs35106420), T659A, T727A, and N693K (rs734644) to be pathogenic R533Q (rs35106420) to be a benign mutation (Table 2a).

**Table 2.** (**a**) Pathogenicity scores for ADGLR3 nsSNPs using different in-silico tools. Scores highlighted in bold indicate that variants are likely to be pathogenic, (b) Prediction of protein folding free energy changes using I-Mutantv 2.0 and Mupro, Scores highlighted in bold suggest that amino acid changes affect protein stability.

(a) SNP	Nucleic acid change	Amino Acid change	MutPred-2	PANTHER-PSEP	PhD-SNP	PolyPhen 2.0	PROVEAN	SIFT
rs35106420	2143 C**G**G→C**A**G	R465Q	0.086	**455**	4	**0.787**	− 1.166	**0.06**
	2347 C**G**G→C**A**G	R533Q	0.089	**455**	4	**0.942**	**− 2.989**	0.05
rs61747658	2724 **A**CG→**G**CG	T659A	0.099	**220**	9	0.001	− 1.197	**0.26**
	2928 **A**CG→**G**CG	T727A	0.103	**220**	1	0.001	− 1.308	**0.24**
rs734644	2828 AA**T**→AA**A**	N693K	0.337	**220**	7	**0.917**	− 0.849	**0.3**

(b) SNP	Nucleic acid change	Amino acid change	I-Mutantv 2.0	Mupro				
rs35106420	2143 C**G**G→C**A**G	R465Q	**−1.181**	**− 1.206**				
	2347 C**G**G→C**A**G	R533Q	**− 1.181**	**− 1.206**				
rs61747658	2724 **A**CG→**G**CG	T659A	**− 0.45**	**− 0.534**				
	2928 **A**CG→**G**CG	T727A	**− 0.45**	**− 0.534**				
rs734644	2828 AA**T**→AA**A**	N693K	0.5	**− 1.476**				

### Prediction of protein stability

We applied I-Mutant 2.0 and Mupro to predict the effects of amino acid substitution caused by nsSNPs on protein stability and the ADGRL3 protein (NP_001877.1) information to compare the effects of mutant and wild-type amino acids on protein folding free energy (Table 2b). Among the changes produced by the nsSNPs (Table 2a), I-Mutant 2.0 predicted that the amino acid substitutions resulted in decreasing protein-free energy in four of these mutations. Furthermore, MUpro predicted that all mutations led to a decrease in protein stability and that the free energy of 105 protein sites would decrease after the mutation of amino acids, resulting in protein stability decline (ΔΔG < 0; Table 2b). Thus, the R465Q (rs35106420), R533Q (rs35106420), and N693K (rs734644) scores were below − 1, indicating that the stability of protein decreased abruptly as a consequence of non-synonymous changes (Table 2b).

### Evolutionary conservation analysis and protein structure analysis

**Figure 2:**
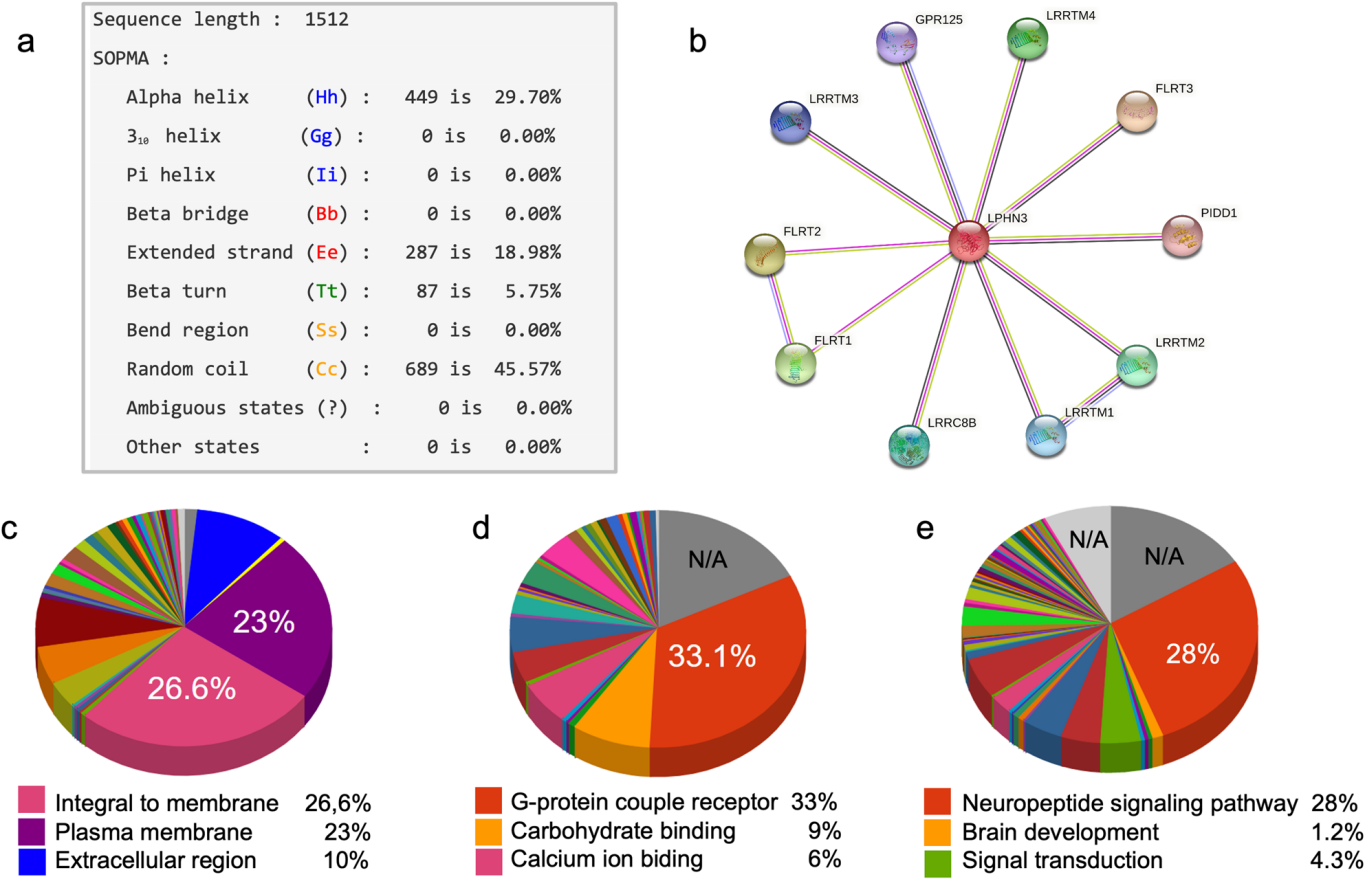
Results of the (**a**) 3D structural analysis of ADGRL3 using SOPMA, (**b**) protein network interaction using STRING, and gene ontology annotations of *ADGRL3* in terms of (**c**) cellular component, (**d**) molecular function, and (**e**) biological process.

ConSurf identified the R533Q (rs35106420) change produced as an evolutionarily conserved position with a score of nine. Using SOPMA, the secondary structure of the ADGRL3 protein was predicted (Fig. 2a). ADGRL3 is composed of 1512 amino acids arranged in four secondary structures; 449 amino acids in the alpha-helix (accounting for 29.70%), 287 amino acids in the extended strand (accounting for 18.98%), 87 amino acids in the beta-turn (accounting for 5.75%), and 689 amino acids in the random coil (accounting for 45.57%) (Fig. 2a).

### ADGRL3 protein structure

#### Protein–protein interactions

STRING predicted interactions of the most studied domain regions of ADGRL3 with Leucine-rich repeat transmembrane protein, Leucine-Rich Repeat Transmembrane Neuronal protein, and members, including FLRT3, FLRT2, FLRT1, LRRTM4, LRRTM2, LRRC8B, LRRTM1, LRRTM3, GPR125, and GPR123 (Fig. 2b).

#### Gene ontology, molecular function, and subcellular localization

Analysis using the CELLO2GO webserver showed that ADGRL3 protein is integral to the membrane (Fig. 2c). *ADGRL3* was validated as a G-protein coupled receptor (Fig. 2d). Furthermore, ADGLR3 is predicted to be involved in biological processes related to neuropeptide signaling and signal transduction (Fig. 2e). Complementary analysis of metabolic pathways involving the Hormone receptor domain (HRM PF02793.22) using the SMART genomic protein database and KEGG orthologous groups showed that this domain is mainly involved in neuroactive ligand-receptor interactions (90.18%; KEGG ID: map04080), followed by long-term depression (7.14%; KEGG ID: map04730) and p53 signaling pathway (2.68%; KEGG ID: map04115).

#### Domain region associated with nsSNPs

Amino acid substitution caused by nsSNP showed two specific regions: (1) the HormR domain, present in hormone receptors and found in the extracellular part of some of the secretin-like (family 2) GPCRs, including the calcitonin receptor, corticotropin-releasing factor receptor 1, diuretic hormone receptor, glucagon-like peptide 1 receptor, and parathyroid hormone peptide receptor (Fig. 1a in green); and (2) the GPCR-Autoproteolysis INducing (GAIN) domain, which is a domain of alpha-helices and beta-strands that is found in cell-adhesion GPCRs and precedes the GPS motif where the autoproteolysis occurs (Fig. 1a in black). The ADGRL3 GAIN domain includes both the GPS and GAIN in cell-adhesion GPCRs and is the functional unit for autoproteolysis. The GPS motif at the end of the GAIN domain is well conserved in primitive ancestor organisms. Furthermore, the full GAIN-GPS is conserved in all cell-adhesion GPCRs and PKD1-related proteins^21^ (Fig. 1).

#### ADGRL3 protein domain architecture analysis

We identified 114 sequences using Gal_Lectin, OLF, GAIN, GPS, 7tm_2, and Latrophilin (see Fig. 1 for more details). The family HRM domain (PF02793.22) showed ten different molecular interactions with other protein domains, including the Glucagon hormone family (*n* = 1), Hormone receptor domain, Immunoglobulin V-set domain, Receptor activity modifying family (*n* = 1) (calcitonin-receptor-like receptor), Secretin receptor family (*n* = 1), Parathyroid hormone family, Glucagon hormone family (*n* = 2), Secretin receptor family (*n* = 2), Receptor activity modifying family (*n* = 2) and Corticotropin-releasing factor family (*n* = 1).

#### Analysis of the ADGRL3 HRM domain

**Figure 3:**
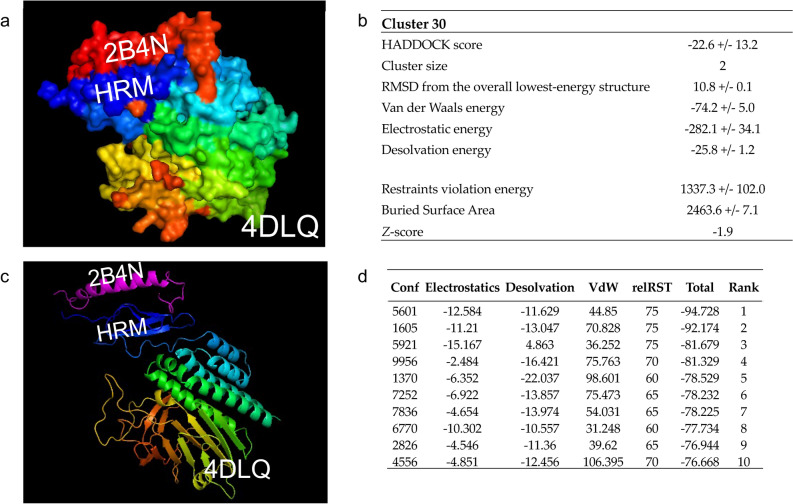
Binding residues corresponding to the 4DLQ and 2B4N proteins. (**a**) HADDOCK diagrammatic illustration of the best-docked complex of 4DLQ–2B4N (Rank 1). (**b**) Scoring functions for the best-docked complex, 4DLQ–2B4N. The *Z*-score indicates how many standard deviations from this cluster’s average are in terms of the score^103^. (**c**) 3D protein structure for the 4DLQ–2B4N complex was identified as the best-docked complex via pyDock (Rank 1). (**d**) Electrostatics, desolvation energy, limited Van der Waals (VdW) contribution scores and restraint-based scoring (relRST) were obtained by the pyDock scoring function. RMDS: Root Mean Square Deviation in Å.

We identified 86 instances of the ADGLR3 HRM domain in the Protein Data Bank (PDB; https://www.rcsb.org/). We selected a PDB-referenced crystal structure containing these domains to study the interaction of both HRM and GAIN domains with other molecules. After conducting a protein sequence similarity analysis using protein–protein BLAST for the ADGRL3 HRM region, we used the 4DLQ, Crystal structure of the GAIN, and HormR domains of CIRL 1/Latrophilin 1 (CL1) from PDB. Next, we applied the Family HRM (PF02793) information of molecules interaction and used the PyMOL molecule alignment feature to determine potential interactions with the ten families from the HRM (PF02793) interactome. We found that the Gastric inhibitory polypeptide (GIP), also known as a Glucose-dependent Insulinotropic Polypeptide (also abbreviated as GIP), aligned with the HRM domain differently (Fig. 3a) than with other molecules from the HRM (PF02793) interactome.

#### Protein–protein docking between HRM and GIP

The 4DLQ Crystal structure of the GAIN and HRM domains of CIRL 1/Latrophilin 1 (CL1)^21^ scored the best similarity with the ADGRL3 region involved in our studies (data not shown). In addition, the family domain description Glucagon/GIP/secretin/VIP (i.e., GIP) was identified as the potential protein interacting with this region. The glucose-dependent insulinotropic polypeptide (GIP) is an incretin hormone that stimulates insulin secretion after ingestion of food. The 2B4N GIP structure was determined by NMR spectroscopy and is characterized by an α-helical motif between residues Ser11 and Gln29^43^.

Before protein–protein docking, we assessed binding residues corresponding to the 4DLQ and 2B4N by CASTp via HADDOCK. HADDOCK clustered 63 structures in 30 clusters, representing 31% of the water-refined models assessed by HADDOCK; the statistics of the top 10 clusters showed cluster 30 as the top cluster most reliable (Rank 1; Fig. 3b). Using pyDock and pyDockWEB, we mapped the 3D coordinates of 4DLQ and 2B4N, corresponding to two hypothetical interacting proteins. pyDockWEB returned the best rigid-body docking orientations generated by FTDock, which was evaluated by pyDock scoring functions after 10.000 replications. As shown in Fig. 3c, the 4DLQ and 2B4N are the best-docked complexes for the ADGRL3 region where the nsSNPs are located (Rank 1; Fig. 3d).

**Figure 4:**
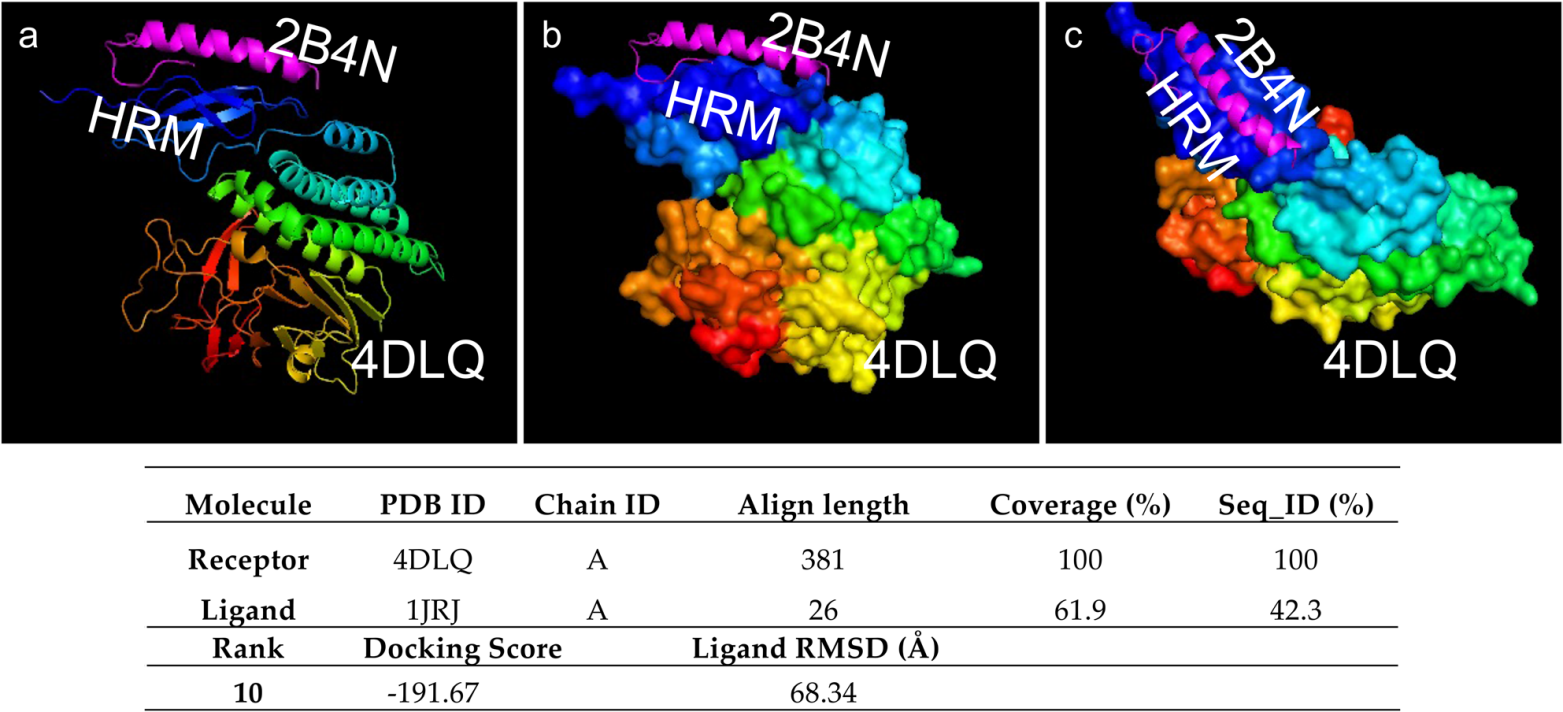
Results of protein–protein docking conducted in HDOCK. Note the three views of 4DLQ (HRM domain) and 2B4N (GIP) in navy blue and magenta, respectively. (**a**) β-helixes and α-helixes complex. (**b**) Surface 4DLQ view with 2B4N α-helix. (**c**) HRM domain pocket where 2B4N (GIP) is predicted to dock. Scoring functions are reported at the bottom. RMDS: Root Mean Square Deviation in Å.

**Figure 5:**
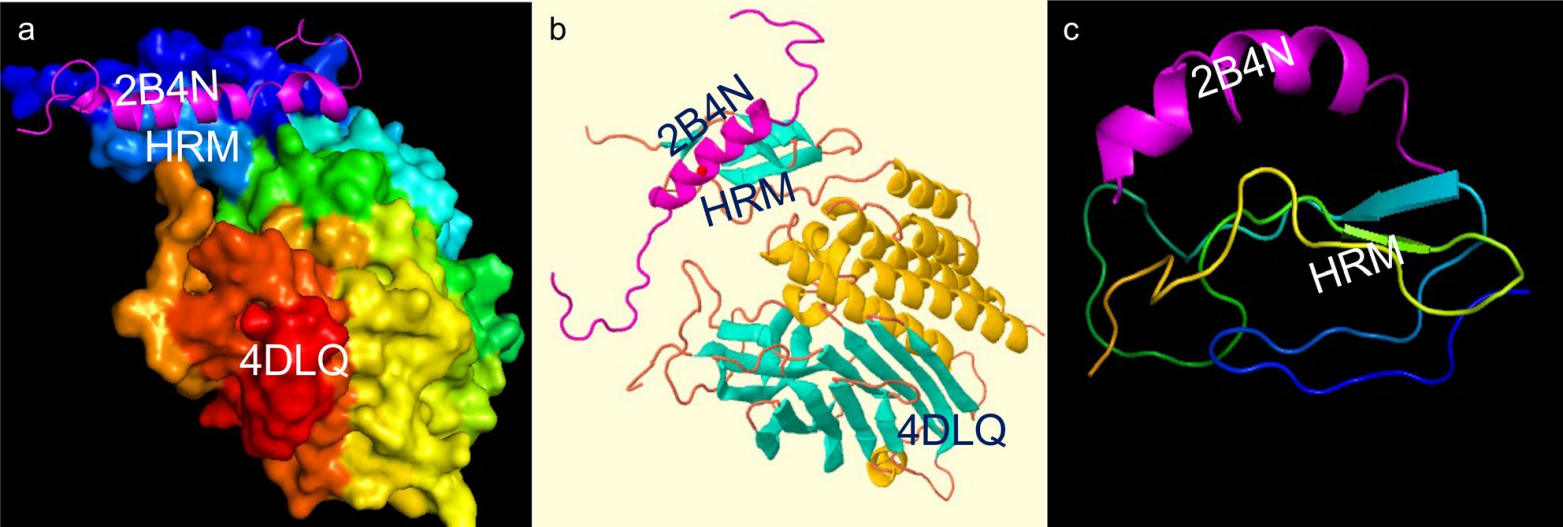
Results of protein–protein docking via (**a**) ClusPro, (**b**) FRODOCK, and (**c**) KBDOCK. Here, 4DLQ (HRM domain) and 2B4N are shown in navy blue in magenta, respectively. Note the reproducibility of the protein–protein docking results between these two molecules.

Results for the protein–protein docking conducted via HDOCK are presented in Fig. 4. We tested the potential protein–protein interaction between HRM and GIP polypeptide using three different in-silico approaches (Fig. 5). Table 2S in the Supplementary Material summarizes the scoring functions assessing the protein–protein interactions between ADGRL3, 4DLQ (HRM domain), and GIP, 2B4N. Overall, all different protein–protein docking tools demonstrate a reproducible interaction between 4DLQ, ADGRL3 HRM domain, and the 2B4N, GIP peptide, with prediction scores between the first ten ranking models with maximum docking scores.

As complementary analyses, we used the AlphaFold2^44,45^ prediction tool on the entire ADGRL3 protein (Fig. 1S, Supplementary Material). We identified regions with a different per-residue confidence score (pLDDT). In particular, the main known domain regions presented very high pLDDT (> 70), while others showed a residue confidence score < 50, which suggests they may be unstructured in isolation^46^. Similarly, AlphaFold2 prediction analysis of the mutated HormR- showed that both regions with pLDDT score higher than 70–90 and regions < 50 residue confidence score, in addition to significant 3D structural changes (Fig. 3S, Supplementary Material).

## Discussion

Candidate gene analyses of single nucleotide polymorphism (SNPs) linked to ADHD have identified variants that have a negligible effect, which still explains only a limited proportion of ADHD variability. Thus, understanding the potential effect of these "small" effects on ADHD etiology is essential. Synonymous SNPs (sSNPs) harbored with the *ADGRL3* gene, previously known as *LPHN3*, have been associated with ADHD through fine-mapping of a genetic linkage region for ADHD observed in the *Paisa* genetic isolate from Antioquia, Colombia^26^. A recent meta-analysis compiled evidence from multiple ADHD genetic studies to support the role of *ADGRL3* sSNPs in ADHD susceptibility in children^3^. Furthermore, *ADGRL3* has been shown to modulate neuronal interaction, axon guidance, and synaptic plasticity, which is highly controlled and orchestrated during CNS development^3,5,17,34^. Using several in-silico tools, here we analyzed the potential pathogenicity effect of non-synonymous SNPs (nsSNPs) harbored in the *ADGRL3* gene, which was previously reported to increase ADHD susceptibility.

Using publicly available databases, we found that 1013 *ADGRL3* nsSNPs have been reported (Table 1S, Supplementary Material), but only three were present in at least two databases and associated with ADHD (rs35106420, rs61747658, and rs734644; Table 1). Next, we applied six pathogenicity prediction software tools (i.e., Mutpred2, PANTHER-PSEP, PhD-SNP, Poly-Phen 2.0, PROVEAN, and SIFT) to assess the pathogenicity of these *ADGRL3* nsSNPs. Our results indicate that marker rs35106420 is predicted to be pathogenic by every analytical tool and shows the highest score in terms of conservation, genetic, and molecular effects, while markers rs61747658 and rs734644, although with lower scores, were predicted to be pathogenic by at least two analytical tools (Table 2a). Interestingly, all nsSNPs were found to significantly decrease ADGRL3 protein stability (Table 2b). Furthermore, evaluation of protein structure and evolutionary conservation determined that the non-synonymous change produced by rs35106420 (R533Q) had the maximum score for highly affecting the conserved amino acid sequence. Therefore, changes in ADGRL3 stability could be accompanied by changes in free energy (Table 2b). This amino acid change indicates ADGRL3 protein stability decreases (ΔΔG < 0; Table 2b).

Further analyses using HOPE showed that when the amino acid changes R465Q (rs35106420) and R533Q (rs35106420) occur for this same marker, the charge of the wild-type (WT) residue will be lost, which can cause loss of interactions with other molecules or residues. Furthermore, the WT and mutant amino acids differ in size, leading to a loss of interactions. Changes in single or multiple amino acids have been shown to cause changes and damage in hydrophobicity, protein folding, central chain tension, and electrostatic force, leading to changes in protein stability. An example is the fragile X mental retardation protein (FMRP), a selective RNA-binding protein implicated in regulating the translation of its mRNA ligands^47^. Developmentally, programmed FMRP expression governs the translation of MAP1B during active synaptogenesis in the neonatal brain. Miss-regulation of the FMRP expression cascade leads to abnormally increased microtubule stability, altering the dynamic organization of the neuronal cytoskeleton and microtubule stability^47^. Our analysis showed that, in the region of the ADGRL3 protein harboring the change induced by the rs35106420 mutation, both R465Q (rs35106420) and R533Q (rs35106420) could play a significant role in affecting ADGRL3 genesis and potentially underly ADHD development of ADHD.

**Figure 6:**
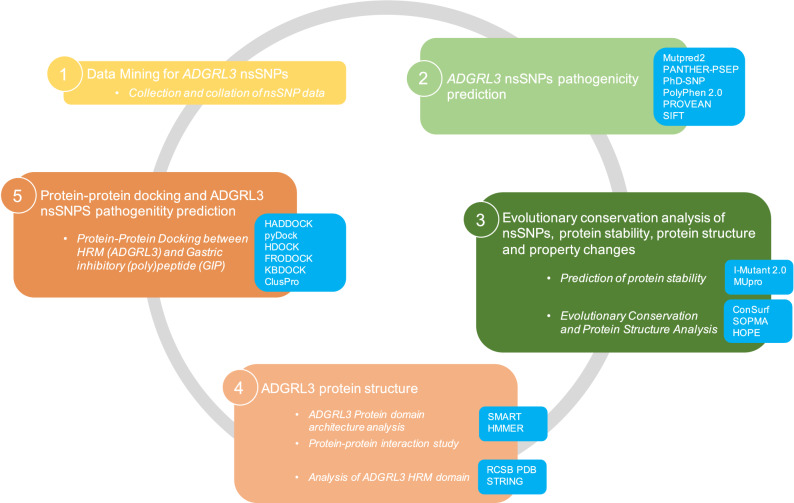
Flow diagram for the in-silico analysis strategy of *ADGRL3* nsSNPs conferring susceptibility to ADHD. Blue squares correspond to in-silico tools used for specific analyses. See “Methods” for more details.

Following our results, we hypothesized that *ADGRL3* nsSNP-related conformational changes located at the HRM domain might disrupt its interaction with GIP downstream signaling. Hence, we applied different protein–protein docking in-silico tools and demonstrated a distinct molecular interaction (Figs. 5 and 6). GIP is expressed in different brain regions, suggesting a potential role as a neurotransmitter or neuromodulator^7,48^. Previously, pleiotropic actions of GIP through the gut-brain axis have also been reported^49^. Furthermore, GIP expression has been correlated to potentiation of the adult hippocampal cells by regulating the neural progenitor cell proliferation in the dentate gyrus of the rat brain^7^.

Glucose-dependent insulinotropic polypeptide receptor (GIPR) increases cAMP, leading to Ca^+^ influx and activation of PKA-CREB signal transduction in β-pancreatic cells^50^. Similarly, GIP analogs have been shown to activate cAMP-PKA-CREB pathway to induce dopaminergic neuron survival^51^. Moreover, GIPR activation has been shown to induce antiapoptotic signaling via p38 MAPK and JNK signaling^52^. During neural development, GIP and GIPR are highly expressed in the internal granule layer (IGL) and the actively divided external granule layer (EGL) within the cerebellum, correlated with their role in neuronal differentiation^53^. Similarly, hippocampal progenitor cells have shown robust GIP expression, driving the differentiation and neurogenesis of neurons and glial cells. Conversely, hippocampal neurogenesis is significantly reduced in the GIP^-/-^ mouse^7^. Furthermore, a recent study found a correlation between GIP and GIPR and the metabolic control by the CNS, as specific CNS deletion of the GIPR showed decreased body weight and improved glucose metabolism^54^. There is also a correlation between glucose metabolic complications and ADHD, such as an increased risk of obesity/overweight^55^.

To shed light on our hypothesis about the molecular relation between ADGRL3 (HRM domain) and GIP signaling disruption linked to ADHD, we also explored key pathways that could play a critical role in ADHD pathophysiology. Recent evidence has linked Wnt signaling and ADHD pathophysiology^56^, with Wnt/β-catenin signaling a critical player throughout all stages of brain development. Consequently, many neurological disorders are associated with the Wnt signaling pathway^57^. Specifically, the delay hypothesis in which dysregulations of this pathway in a time-dependent manner could lead to neurodevelopmental delays, resulting in the ADHD phenotype^56^. Several studies have demonstrated the link between Wnt and GIP pathways^58–61^. In addition, *gip* mRNA expression was stimulated by the Wnt signaling cascade; an induction using lithium or Wnt/β-cat signaling enhanced GIP production by entero-endocrine cells through a conserved T cell factor (TCF) binding site within the proximal region of the *gip* promoter^62^. Therefore, we hypothesize that dysregulation of the GIP signaling downstream of the Wnt pathway could lead to neurodevelopmental delays and result in the ADHD phenotype.

Analyses of ADGRL3 protein structure allowed the identification of different predicted domains (Fig. 3 and Table 2S). We found that the *ADGRL3* nsSNPS were located within the hormone receptor domain (HRM) and the GPCR-Autoproteolysis Inducing (GAIN) domains^21^. Although the GAIN, Gal Lectin (Galactosa binding lectin), and OLF (Olfactomedin-like) domains of AGDRL3 have been previously studied^63,64^, little is known about the HRM domain. Here we show, through different in-silico analyses, that HRM and GAIN domains play a significant role in the ADGRL3 function. Indeed, previous functional studies showed that the GAIN domain is necessary and sufficient for autoproteolysis, suggesting that the GAIN domain plays a role in fine-tuning the chemical environment in the GPS to catalyze peptide bond hydrolysis^21^. This domain represents an evolutionarily autoproteolytic fold relevant for GPCR signaling and potentially be related to several human disorders^21^. Conversely, the HRM domain of ADGRL3 has not been studied previously. However, our in-silico analyses showed that nsSNPs within this region might produce changes in ADGRL3 protein.

In addition, analyses of the ADGRL3 protein domain and architecture identified that the Family HRM domain (PF02793) showed ten different molecular interactions with other protein domains (Fig. 4a). Next, we used in-silico tools to examine potential molecular interactions between the HRM domain and different candidate molecules. Because protein–protein interactions play a crucial role in several cellular processes such as signal transduction, protein expression regulation, and DNA replication, providing insight into these molecular interactions is paramount to deciphering disease mechanisms at the molecular level. Nevertheless, only a small number of the 3D structures of protein–protein complexes have been determined experimentally and deposited into the released databases such as Protein Data Bank (PDB; https://www.rcsb.org/). Computational methods such as protein–protein docking have been increasingly applied to the structural prediction of macromolecular assemblies. Thus, we sought to analyze HRM molecular interactions previously described using different protein–protein docking in-silico tools (Figs. 4 and 5). These analyses predicted a good molecular alignment within the first rank positions and docking scores (Fig. 4b and Table 2S), corresponding to a small polypeptide (i.e., gastric inhibitory polypeptide or GIP) interacting with the HRM region of ADGRL3. In particular, docking analysis using HDOCK showed the structure prediction for protein–protein docking between HRM and GIP (Fig. 4), and the interaction between HRM and GIP was further validated using three different protein–protein docking suites (Fig. 5). This peptide, also known as a glucose-dependent insulinotropic polypeptide (also referred to as GIP), is an inhibiting hormone of the secretin family of hormones that has been shown to stimulate insulin secretion^65^. Analysis by cDNA arrays comparing the gender differential expression in the neurogenesis process between adult rats showed that the Hippocampal expression of the gene-encoding GIP varied enormously in parallel with cell-proliferation rates in the hippocampal dentate gyrus (DG) of adult rats^7^. It has been previously demonstrated that adult male and female rats from the Sprague Dawley and spontaneously hypertensive strains have a marked difference in proliferation rates of cells in the hippocampal DG^66^.

ADHD has been reported more often in males than females, with population and clinical studies showing male:female ratios of 4:1 and 9:1, respectively^67–69^. In addition, individuals with ADHD show macro-anatomical changes in multiple brain regions resulting from disrupted neurodevelopmental mechanisms. Hoogman et al.^70^, through the ENIGMA collaboration, one of the most extensive imaging meta-analyses to date, demonstrated that individuals with ADHD have significantly smaller brain volumes than healthy controls in the accumbens, caudate, putamen, amygdala, and hippocampus^70,71^. In-silico prediction of protein–protein interactions between GIP and the HRM region of ADGRL3, showed that these interactions could be affected by mutations in the HRM region and be a potential moiety interaction target to explore the mechanisms underpinning the increased risk of developing ADHD. Expression levels of ADGRL3 human mRNA are higher in the cerebral cortex, amygdala, and hippocampus (Fig. 2S, Supplementary Material), which are brain regions that modulate high-level brain-controlled functions such as maintaining attention, executive function, and organization^70,71^. An imbalance within these brain regions might cause inattention, problems with organization, and impaired executive functioning^72^.

Evidence indicates that GIP strongly stimulates insulin secretion in the presence of elevated glucose^65^. This phenomenon of higher insulin secretion in response to oral glucose compared to intravenous glucose at the same plasma glucose level is called the incretin effect^73^. GIP is considered the most potent incretin hormone; along with glucagon-like peptide-1 (GLP-1), it contributes to 25 to 70% of the postprandial insulin response^74^. GIP is expressed in the pancreas as well as the gut, adipose tissue, heart, pituitary, and inner layers of the adrenal cortex, and it is also expressed in several key brain regions, including the cerebral cortex, hippocampus, and olfactory bulb^75^, suggesting that GIP may have previously undescribed actions. Interestingly, PET scans showed that cerebral glucose metabolism is 8.1% lower in individuals with ADHD than in healthy controls^76^. Previous studies have suggested that GIP plays an essential role in glucose metabolism. Specifically, GIP's inadequate response has been linked to diabetes mellitus (DM)^77^. Moreover, analysis of longitudinal levels of fasting GIP during pregnancy has shown that lower levels of GIP may play a role in the increased risk of gestational diabetes mellitus (GDM) and altered glucose regulation after pregnancy^78^.

An association between ADHD and metabolic disorders such as diabetes has recently been reported. In particular, a population-based study analyzing ~ 15,000 Swedish individuals born after their parents were diagnosed with type 1 diabetes (T1D) showed a significantly increased risk of ADHD (Hazard Ratio [HR] = 1.29, 95% confidence interval [CI] = 1.15–1.42)^79^. In this study, maternal T1D was associated with an enhanced risk of ADHD (HR = 1.35, 95%CI = 1.18–1.55) compared with paternal T1D (HR = 1.20, 95% CI = 1.03–1.41). Hence, parental history of T1D was associated with a 29% increased risk of being diagnosed with ADHD. Another study showed that the three types of diabetes during pregnancy (T1D, T2D, and GDM) were associated with offspring ADHD^80^. Similarly, association analyses have shown that adult individuals with ADHD have a higher risk for diabetes than adults without ADHD^81^. Furthermore, it has been shown that diabetic treatment may improve ADHD symptoms during adulthood. Metformin, a prescription drug to treat diabetes, has recently been linked to improving cognitive and behavioral domains in adults with fragile X syndrome^82^ and induces neurogenesis in the adult mouse CNS^6^.

It is quite intriguing that the *ADGLR3* HRM domain has not been previously associated with increased ADHD risk. Protein–protein interaction analyses using different protein–protein docking computational tools predicted a good molecular alignment between ADGRL3 and GIP within the first rank positions and docking scores (Table 2S), suggesting that this interaction may represent a vital ADHD etiological factor that could modulate neuroplasticity, neurotransmission, and cell proliferation during embryogenesis^83–86^, a significant event related to ADHD, and suggest that GIP receptors play an essential role in cognition, neurotransmission, and cell proliferation.

In summary, we performed a series of in-silico analyses of nsSNPs to understand best the association between ADGRL3 and ADHD risk. These analyses showed: (i) potential pathogenic effects of these nsSNP mutations, (ii) potential interactions between specific regions of *ADGRL3* harboring these nsSNP changes, *i.e.,* the HRM domain and the Gastric inhibitory polypeptide (GIP) of the secretin family that plays a crucial role in neuronal biogenesis and neural cell division. Overall, our findings provide valuable information about the pathogenicity and evolutionary conservation and protein stability, protein structure, and property changes of ADGRL3 as a consequence of these non-synonymous changes. Thus, our in-silico findings not only suggest a link between the disrupted standard and wild ADGRL3 structure and its metabolic regulation consequences but grant a further exploration of in vitro experiments to define and evaluate the possible therapeutic possibility for both children and adults with ADHD.

## Methods

To identify *ADGRL3* nsSNPs previously reported to confer susceptibility to ADHD and study their effect on ADGRL3 stability, function, and regulation, we followed a five-step in-silico approach (Fig. 6).

### Data mining of ADGRL3 nsSNPs

We performed data mining of nsSNPs based on four databases: the database for single nucleotide polymorphism (dbSNP, RRID: SCR_002338, https://www.ncbi.nlm.nih.gov/projects/SNP/)^87^, the nsSNPdpe database (nsSNP database of functional effects, https://www.rostlab.org/services/snpdbe/), and DisGeNET database (RRID: SCR_006178, https://www.disgenet.org/)^88^. We also scrutinized the results of case/control- and family-based genetic studies on ADHD to retrieve data on nsSNPs conferring susceptibility to the disorder^26,35,37^.

### ADGRL3 nsSNPs pathogenicity prediction

In-silico pathogenicity prediction of the retrieved nsSNPs was evaluated using six different software tools, including MutPred2 (RRID: SCR_010778, http://mutpred.mutdb.org/)^89^, PANTHER^90^, PSEP (RRID: SCR_005145, http://pantherdb.org/tools/csnpScoreForm.jsp)^91^, PhD-SNP (RRID: SCR_010782, http://snps.biofold.org/phd-snp/phd-snp.html)^92^, PolyPhen 2.0 (RRID: SCR_013189, http://genetics.bwh.harvard.edu/pph2/)^93^, PROVEAN (RRID: SCR_002182, http://provean.jcvi.org/index.php)^94^, and SIFT (RRID: SCR_012813, http://sift.bii.a-star.edu.sg/)^95^. In the following paragraphs we will briefly describe each tool.

MutPred2 is a Machine Learning-based method and software package that integrates genetic and molecular data to evaluate the potential pathogenicity of amino acid substitutions probabilistically. MutPred2 tests various structural and functional properties, including secondary structure, signal peptide, transmembrane topology, catalytic activity, macromolecular binding, PTMs, metal binding, and allostery. When using MutPred2, an nsSNP with a score > 0.5–1 will be considered pathogenic^89^.

The PANTHER-PSEP applied the measurements on millions of years (my) of conservation of a specific SNP position (measured in my of conservation). Thus, a score > 450my is marked as probably damaging, a score of > 200my is marked as possibly damaging, and a score of < 200my is marked as probably benign^91^.

PhD-SNP is an in-silico tool based on support vector machines (SVMs) and a binary classifier based on a Gradient Boosting algorithm that uses a protein sequence and predicts whether a new phenotype derived from an nsSNP can be related to a genetic disease in humans using sequence and phylogenetic conservation information^92^. PhD-SNP has been trained on thousands of variants extracted from databases collecting pathogenic mutations.

PolyPhen 2.0 compares the wild-type (ancestral, normal) allele and the corresponding property of the mutant (derived, disease-causing) nsSNPs allele using straightforward physical and comparative considerations, i.e., This prediction is based on several features comprising the sequence, phylogenetic and structural information characterizing the substitution^93^. For a given amino acid substitution in a protein, PolyPhen-2 extracts various sequence and structure-based features of the substitution site and feeds them to a probabilistic classifier for a given amino acid substitution in a protein. Mutations with scores > 0.50 are predicted to be pathogenic.

PROVEAN^94^ and SIFT^95^ compare sequence homology-based in-a-silico tools. While mutations with a pathogenicity score > − 2.5 are predicted to be deleterious or pathogenic in the former, in SIFT, mutations with scores > 0.05 are predicted as pathogenic.

### Evolutionary conservation analysis of nsSNPs, protein stability, structure, and property changes

Molecular protein stability assesses how changes in specific amino acids can affect the function and activity of biological molecules^41,96^. We used I-Mutant 2.0 (https://folding.biofold.org/i-mutant/i-mutant2.0.html)^92^ and MUpro (http://mupro.proteomics.ics.uci.edu/)^96,97^ to evaluate free energy of protein unfolding in a mutated protein. This in-silico technique identifies changes in protein stability and predicts changes in stability by evaluating thermodynamic free energy changes (ΔΔG) and the direction of the change after a single point mutation of protein. Thus, positive values of ΔΔG indicate stabilization while negative values indicate destabilization^97,98^.

Protein evolutionary conservation is crucial for determining potential structural and functional modifications by mutational changes^97,99^. To analyze the evolutionary conservation of amino acids, we used ConSurf (http://consurf.tau.ac.il); scores between 7 and 9 were considered evolutionary conservative amino acids.

### ADGRL3 protein structure

To predict the secondary structure of proteins^99^, we used the self-optimized prediction method (SOPMA; https://npsa-prabi.ibcp.fr/cgi-bin/npsa_automat.pl?page=npsa_sopma.html), which collects structural information from calculations on the 3D protein structure, sequence annotations in UniProt and prediction from the Reprof software, and HOPE (http://www.cmbi.ru.nl/hope/input/). HOPE combines this information to give insights into the effect of a specific mutation on the protein structure and assess the physical and chemical properties of the SNPs^100^. The STRING (https://string-db.org/)^101^ database explored protein–protein interaction networks and annotated ADGRL3 protein interaction with other proteins. We also applied the Simple Modular Architecture Research Tool (SMART; http://smart.embl.de)^101^ and used the HMMER web server (http://www.ebi.ac.uk/Tools/hmmer) for the identification and annotation of protein domains and the analysis of protein domain architectures. HMMER is a free-to-use service that provides fast searches against widely used sequence databases and profiles Hidden Markov Model (HMM) libraries via the HMMER software suite (http://hmmer.org)^102^.

### Protein–protein docking and ADGRL3 nsSNPs pathogenicity prediction

Structurally protein–protein interactions have been experimentally determined and placed at the Protein Data Bank (PDB; https://www.rcsb.org/). We aimed to evaluate the potential interacting partners of the HRM domain, which was the domain within ADGRL3 showing a risk correlation with ADHD, specifically the 4DLQ Crystal structure of the GAIN and HormR domains of CIRL 1/Latrophilin 1 (CL1)^21^. This structure scored the best similarity with the region involved in our studies (data not shown).

To characterize the molecular interaction, we applied protein–protein docking using different models. We first used High Ambiguity Driven protein–protein DOCKing (HADDOCK; https://wenmr.science.uu.nl/haddock2.4/)^103^. A second approach included using pyDockWEB (https://life.bsc.es/pid/pydockweb/default/index), a web server for the structural prediction of protein–protein docking. By giving the 3D coordinates of the interacting proteins, the program returns the best rigid-body docking orientations, which are generated by FTDock and assessed by pyDock^104^ scoring function using electrostatics, desolvation energy, and little van der Waals contribution. pyDock is written in Python and uses the MMTK set of Python libraries for parsing PDB files, calculating AMBER charges, and other common tasks^105^.

We also used HDOCK (http://huanglab.phys.hust.edu.cn/software/hdocklite/)^106^, which is a docking program that first samples the putative binding modes between two proteins through a fast Fourier transform (FFT)-based global search method, and then evaluated the sampled binding modes with an improved iterative knowledge-based scoring function for protein–protein interactions.

Additionally, we used ClusPro (https://cluspro.org)^107^, an in-silico tool for protein–protein docking. ClusPro allows the removal of unstructured protein regions, application of attraction or repulsion, accounting for pairwise distance restraints, construction of homo-multimers, consideration of small-angle X-ray scattering (SasaXS) data, and location of heparin-binding sites. Six different energy functions can be used, depending on the type of protein. Docking with each energy parameter set results in ten models defined by centers of highly populated clusters of low-energy docked structures^107^. We also applied FRODOCK (http://frodock.chaconlab.org/)^108^.

Finally, we applied KBDOCK (https://kbdock.loria.fr/index.php)^109^, a 3D database system that defines and spatially clusters protein binding sites for knowledge-based protein docking. KBDOCK extracts protein domain-domain interaction (DDI) and domain-peptide interaction (DPI) information from the PDB using the PFAM domain classification in order to analyze the spatial arrangements of DDIs and DPIs by the Pfam family and to propose structural templates for protein docking^109^.

## Supplementary Information

Supplementary Information 1.

Supplementary Table S1.

## Data Availability

The datasets used and analyzed during the current study are available from the corresponding author on reasonable request. We performed data mining of non-synonymous SNPs (nsSNPs) based on dbSNP (RRID: SCR_002338, https://www.ncbi.nlm.nih.gov/projects/SNP/) and DisGeNET database (RRID: SCR_006178, https://www.disgenet.org/). In-silico pathogenicity prediction of the retrieved nsSNPs was evaluated using MutPred2 (RRID: SCR_010778, http://mutpred.mutdb.org/), PANTHER (RRID: SCR_005145, http://pantherdb.org/tools/csnpScoreForm.jsp), PhD-SNP (RRID: SCR_010782, http://snps.biofold.org/phd-snp/phd-snp.html)^92^, PolyPhen 2.0 (RRID: SCR_013189, http://genetics.bwh.harvard.edu/pph2/)^93^, PROVEAN (RRID: SCR_002182, http://provean.jcvi.org/index.php)^94^, and SIFT (RRID: SCR_012813, http://sift.bii.a-star.edu.sg/).

## References

[1] Acosta MT, Arcos-Burgos M, Muenke M (2004). Attention deficit/hyperactivity disorder (ADHD): Complex phenotype, simple genotype?. Genet. Med..

[2] Arcos-Burgos M, Muenke M (2010). Toward a better understanding of ADHD: LPHN3 gene variants and the susceptibility to develop ADHD. Atten. Defic. Hyperact. Disord..

[3] Bruxel EM (2021). Meta-analysis and systematic review of ADGRL3 (LPHN3) polymorphisms in ADHD susceptibility. Mol. Psychiatry..

[4] Cervantes-Henriquez ML (2021). Machine learning prediction of ADHD severity: Association and linkage to ADGRL3, DRD4, and SNAP25. J. Atten. Disord..

[5] Faraone SV, Banaschewski T, Coghill D, Zheng Y, Biederman J, Bellgrove MA (2021). The World Federation of ADHD International Consensus Statement: 208 Evidence-based conclusions about the disorder. Neurosci. Biobehav. Rev..

[6] Wang J, Gallagher D, DeVito LM, Cancino GI, Tsui D, He L (2012). Metformin activates an atypical PKC-CBP pathway to promote neurogenesis and enhance spatial memory formation. Cell Stem Cell.

[7] Nyberg J, Anderson MF, Meister B, Alborn AM, Ström AK, Brederlau A (2005). Glucose-dependent insulinotropic polypeptide is expressed in adult hippocampus and induces progenitor cell proliferation. J. Neurosci..

[8] Poelmans G, Pauls DL, Buitelaar JK, Franke B (2011). Integrated genome-wide association study findings: Identification of a neurodevelopmental network for attention deficit hyperactivity disorder. Am. J. Psychiatry..

[9] Purper-Ouakil D, Lepagnol-Bestel AM, Grosbellet E, Gorwood P, Simonneau M (2010). Neurobiology of attention deficit/hyperactivity disorder. Med. Sci..

[10] Faraone SV, Larsson H (2019). Genetics of attention deficit hyperactivity disorder. Mol. Psychiatry..

[11] Demontis D, Walters RK, Martin J, Mattheisen M, Als TD, Agerbo E (2019). Discovery of the first genome-wide significant risk loci for attention deficit/hyperactivity disorder. Nat. Genet..

[12] Granier S, Kobilka B (2012). A new era of GPCR structural and chemical biology. Nat. Chem. Biol..

[13] Bassilana F, Nash M, Ludwig MG (2019). Adhesion G protein-coupled receptors: Opportunities for drug discovery. Nat. Rev. Drug Discov..

[14] Rajagopal S, Rajagopal K, Lefkowitz RJ (2010). Teaching old receptors new tricks: Biasing seven-transmembrane receptors. Nat. Rev. Drug Discov..

[15] Krishnan A, Nijmeijer S, de Graaf C, Schiöth HB (2016). Classification, nomenclature, and structural aspects of adhesion GPCRs. Handb. Exp. Pharmacol..

[16] Röthe J, Thor D, Winkler J, Knierim AB, Binder C, Huth S (2019). Involvement of the adhesion GPCRs latrophilins in the regulation of insulin release. Cell Rep..

[17] Martinez AF, Abe Y, Hong S, Molyneux K, Yarnell D, Löhr H (2016). An ultraconserved brain-specific enhancer within ADGRL3 (LPHN3) underpins attention-deficit/hyperactivity disorder susceptibility. Biol. Psychiatry..

[18] Moreno-Salinas AL, Avila-Zozaya M, Ugalde-Silva P, Hernández-Guzmán DA, Missirlis F, Boucard AA (2019). Latrophilins: A neuro-centric view of an evolutionary conserved adhesion g protein-coupled receptor subfamily. Front. Neurosci..

[19] Hamann J, Aust G, Araç D, Engel FB, Formstone C, Fredriksson R (2015). International union of basic and clinical pharmacology. XCIV. Adhesion G protein-coupled receptors. Pharmacol. Rev..

[20] Zhao J, Deng Y, Jiang Z, Qing H (2016). G protein-coupled receptors (GPCRs) in Alzheimer's disease: A focus on BACE1 related GPCRs. Front. Aging Neurosci..

[21] Araç D, Boucard AA, Bolliger MF, Nguyen J, Soltis SM, Südhof TC (2012). A novel evolutionarily conserved domain of cell-adhesion GPCRs mediates autoproteolysis. EMBO J..

[22] Orsini CA, Setlow B, DeJesus M, Galaviz S, Loesch K, Ioerger T (2016). Behavioral and transcriptomic profiling of mice null for Lphn3, a gene implicated in ADHD and addiction. Mol. Genet. Genomic Med..

[23] Acosta MT, Velez JI, Bustamante ML, Balog JZ, Arcos-Burgos M, Muenke M (2011). A two-locus genetic interaction between LPHN3 and 11q predicts ADHD severity and long-term outcome. Transl. Psychiatry..

[24] Jain M, Velez JI, Acosta MT, Palacio LG, Balog J, Roessler E (2012). A cooperative interaction between LPHN3 and 11q doubles the risk for ADHD. Mol. Psychiatry.

[25] Ribases M, Ramos-Quiroga JA, Sanchez-Mora C, Bosch R, Richarte V, Palomar G (2011). Contribution of LPHN3 to the genetic susceptibility to ADHD in adulthood: A replication study. Genes Brain Behav..

[26] Arcos-Burgos M, Jain M, Acosta MT, Shively S, Stanescu H, Wallis D (2010). A common variant of the latrophilin 3 gene, LPHN3, confers susceptibility to ADHD and predicts effectiveness of stimulant medication. Mol. Psychiatry.

[27] Choudhry Z, Sengupta SM, Grizenko N, Fortier ME, Thakur GA, Bellingham J (2012). LPHN3 and attention-deficit/hyperactivity disorder: Interaction with maternal stress during pregnancy. J. Child psychol. Psychiatry Allied Discipl..

[28] Labbe A, Liu A, Atherton J, Gizenko N, Fortier ME, Sengupta SM (2012). Refining psychiatric phenotypes for response to treatment: Contribution of LPHN3 in ADHD. Am. J. Med. Genet. B Neuropsychiatr. Genet..

[29] Gomez-Sanchez CI, Riveiro-Alvarez R, Soto-Insuga V, Rodrigo M, Tirado-Requero P, Mahillo-Fernandez I (2016). Attention deficit hyperactivity disorder: Genetic association study in a cohort of Spanish children. Behav. Brain Funct..

[30] Bruxel EM, Salatino-Oliveira A, Akutagava-Martins GC, Tovo-Rodrigues L, Genro JP, Zeni CP (2015). LPHN3 and attention-deficit/hyperactivity disorder: A susceptibility and pharmacogenetic study. Genes Brain Behav..

[31] Hwang IW, Lim MH, Kwon HJ, Jin HJ (2015). Association of LPHN3 rs6551665 A/G polymorphism with attention deficit and hyperactivity disorder in Korean children. Gene.

[32] Domené S, Stanescu H, Wallis D, Tinloy B, Pineda DE, Kleta R (2011). Screening of human LPHN3 for variants with a potential impact on ADHD susceptibility. Am. J. Med. Genet. B Neuropsychiatr. Genet..

[33] Martinez AF, Muenke M, Arcos-Burgos M (2011). From the black widow spider to human behavior: Latrophilins, a relatively unknown class of G protein-coupled receptors, are implicated in psychiatric disorders. Am. J. Med. Genet. B Neuropsychiatr. Genet..

[34] Regan SL, Williams MT, Vorhees CV (2021). Latrophilin-3 disruption: Effects on brain and behavior. Neurosci. Biobehav. Rev..

[35] Acosta MT, Swanson J, Stehli A, Molina BS, Martinez AF, Arcos-Burgos M (2016). ADGRL3 (LPHN3) variants are associated with a refined phenotype of ADHD in the MTA study. Mol. Genet. Genomic Med..

[36] Kappel DB, Schuch JB, Rovaris DL, da Silva BS, Müller D, Breda V (2019). ADGRL3 rs6551665 as a common vulnerability factor underlying attention-deficit/hyperactivity disorder and autism spectrum disorder. Neuromolecular Med..

[37] Puentes-Rozo PJ, Acosta-Lopez JE, Cervantes-Henriquez ML, Martinez-Banfi ML, Mejia-Segura E, Sanchez-Rojas M (2019). Genetic variation underpinning ADHD risk in a Caribbean community. Cells.

[38] Bhatnager R, Dang AS (2018). Comprehensive in-silico prediction of damage associated SNPs in Human Prolidase gene. Sci. Rep..

[39] Hossain MS, Roy AS, Islam MS (2020). In silico analysis predicting effects of deleterious SNPs of human RASSF5 gene on its structure and functions. Sci. Rep..

[40] Arcos-Burgos M, Velez JI, Martinez AF, Ribases M, Ramos-Quiroga JA, Sanchez-Mora C (2019). ADGRL3 (LPHN3) variants predict substance use disorder. Transl. Psychiatry..

[41] Banerjee A, Levy Y, Mitra P (2019). Analyzing change in protein stability associated with single point deletions in a newly defined protein structure database. J. Proteome Res..

[42] Persikov AV, Pillitteri RJ, Amin P, Schwarze U, Byers PH, Brodsky B (2004). Stability related bias in residues replacing glycines within the collagen triple helix (Gly-Xaa-Yaa) in inherited connective tissue disorders. Hum. Mutat..

[43] Alaña I, Parker JC, Gault VA, Flatt PR, O'Harte FP, Malthouse JP (2006). NMR and alanine scan studies of glucose-dependent insulinotropic polypeptide in water. J. Biol. Chem..

[44] Jumper J, Evans R, Pritzel A, Green T, Figurnov M, Ronneberger O (2021). Highly accurate protein structure prediction with AlphaFold. Nature.

[45] Senior AW, Evans R, Jumper J, Kirkpatrick J, Sifre L, Green T (2020). Improved protein structure prediction using potentials from deep learning. Nature.

[46] Mirdita, M. *et al.* ColabFold - Making protein folding accessible to all. *bioRXiv* (2022). 10.1038/s41592-022-01488-1 PMC9184281 35637307

[47] Lu R, Wang H, Liang Z, Ku L, O’donnell WT, Li W (2004). The fragile X protein controls microtubule-associated protein 1B translation and microtubule stability in brain neuron development. Proc. Natl. Acad. Sci. USA.

[48] Nyberg J, Jacobsson C, Anderson MF, Eriksson PS (2007). Immunohistochemical distribution of glucose-dependent insulinotropic polypeptide in the adult rat brain. J. Neurosci. Res..

[49] Drucker DJ, Sherman SI, Gorelick FS, Bergenstal RM, Sherwin RS, Buse JB (2010). Incretin-based therapies for the treatment of type 2 diabetes: Evaluation of the risks and benefits. Diabetes Care.

[50] Lu M, Wheeler MB, Leng XH, Boyd AE (1993). The role of the free cytosolic calcium level in beta-cell signal transduction by gastric inhibitory polypeptide and glucagon-like peptide I(7–37). Endocrinology.

[51] Li Y, Liu W, Li L, Holscher C (2016). Neuroprotective effects of a GIP analogue in the MPTP Parkinson's disease mouse model. Neuropharmacology.

[52] Widenmaier SB, Ao Z, Kim SJ, Warnock G, McIntosh CH (2009). Suppression of p38 MAPK and JNK via Akt-mediated inhibition of apoptosis signal-regulating kinase 1 constitutes a core component of the beta-cell pro-survival effects of glucose-dependent insulinotropic polypeptide. J. Biol. Chem..

[53] Paratore S, Ciotti MT, Basille M, Vaudry D, Gentile A, Parenti R (2011). Gastric inhibitory polypeptide and its receptor are expressed in the central nervous system and support neuronal survival. Cent. Nerv. Syst. Agents Med. Chem..

[54] Zhang Q, Delessa CT, Augustin R, Bakhti M, Collden G, Drucker DJ (2021). The glucose-dependent insulinotropic polypeptide (GIP) regulates body weight and food intake via CNS-GIPR signaling. Cell Metab..

[55] Bowling AB, Tiemeier HW, Jaddoe VWV, Barker ED, Jansen PW (2018). ADHD symptoms and body composition changes in childhood: A longitudinal study evaluating directionality of associations. Pediatr. Obes..

[56] Yde Ohki CM, Grossmann L, Alber E, Dwivedi T, Berger G, Werling AM (2020). The stress-Wnt-signaling axis: A hypothesis for attention-deficit hyperactivity disorder and therapy approaches. Transl. Psychiatry..

[57] Noelanders R, Vleminckx K (2017). How Wnt signaling builds the brain: Bridging development and disease. Neuroscientist..

[58] Garcia-Martinez JM, Chocarro-Calvo A, Moya CM, Garcia-Jimenez C (2009). WNT/beta-catenin increases the production of incretins by entero-endocrine cells. Diabetologia.

[59] Jin T, Liu L (2008). The Wnt signaling pathway effector TCF7L2 and type 2 diabetes mellitus. Mol. Endocrinol..

[60] Sun J, Khalid S, Rozakis-Adcock M, Fantus IG, Jin T (2009). P-21-activated protein kinase-1 functions as a linker between insulin and Wnt signaling pathways in the intestine. Oncogene.

[61] Yi F, Sun J, Lim GE, Fantus IG, Brubaker PL, Jin T (2008). Cross talk between the insulin and Wnt signaling pathways: Evidence from intestinal endocrine L cells. Endocrinology.

[62] Chiang YT, Ip W, Jin T (2012). The role of the Wnt signaling pathway in incretin hormone production and function. Front. Physiol..

[63] Jackson VA, Mehmood S, Chavent M, Roversi P, Carrasquero M, Del Toro D (2016). Super-complexes of adhesion GPCRs and neural guidance receptors. Nat. Commun..

[64] Lu YC, Nazarko OV, Sando R, Salzman GS, Li NS, Südhof TC (2015). Structural basis of latrophilin-FLRT-UNC5 interaction in cell adhesion. Structure..

[65] Pederson RA, McIntosh CH (2016). Discovery of gastric inhibitory polypeptide and its subsequent fate: Personal reflections. J. Diabetes Investig..

[66] Perfilieva E, Risedal A, Nyberg J, Johansson BB, Eriksson PS (2001). Gender and strain influence on neurogenesis in dentate gyrus of young rats. J. Cereb. Blood Flow Metab..

[67] Biederman J, Mick E, Faraone SV, Braaten E, Doyle A, Spencer T (2002). Influence of gender on attention deficit hyperactivity disorder in children referred to a psychiatric clinic. Am. J. Psychiatry..

[68] Cuffe SP, Moore CG, McKeown RE (2005). Prevalence and correlates of ADHD symptoms in the national health interview survey. J. Atten. Disord..

[69] Dark C, Homman-Ludiye J, Bryson-Richardson RJ (2018). The role of ADHD associated genes in neurodevelopment. Dev. Biol..

[70] Hoogman M, Bralten J, Hibar DP, Mennes M, Zwiers MP, Schweren LSJ (2017). Subcortical brain volume differences in participants with attention deficit hyperactivity disorder in children and adults: A cross-sectional mega-analysis. Lancet Psychiatry.

[71] Almeida LG, Ricardo-Garcell J, Prado H, Barajas L, Fernández-Bouzas A, Avila D (2010). Reduced right frontal cortical thickness in children, adolescents and adults with ADHD and its correlation to clinical variables: A cross-sectional study. J. Psychiatr. Res..

[72] Plessen KJ, Bansal R, Zhu H, Whiteman R, Amat J, Quackenbush GA (2006). Hippocampus and amygdala morphology in attention-deficit/hyperactivity disorder. Arch. Gen. Psychiatry..

[73] Dupre J, Ross SA, Watson D, Brown JC (1973). Stimulation of insulin secretion by gastric inhibitory polypeptide in man. J. Clin. Endocrinol. Metab..

[74] Kuhre RE, WewerAlbrechtsen NJ, Hartmann B, Deacon CF, Holst JJ (2015). Measurement of the incretin hormones: Glucagon-like peptide-1 and glucose-dependent insulinotropic peptide. J. Diabetes Complications..

[75] Usdin TB, Mezey E, Button DC, Brownstein MJ, Bonner TI (1993). Gastric inhibitory polypeptide receptor, a member of the secretin-vasoactive intestinal peptide receptor family, is widely distributed in peripheral organs and the brain. Endocrinology.

[76] Zametkin AJ, Nordahl TE, Gross M, King AC, Semple WE, Rumsey J (1990). Cerebral glucose metabolism in adults with hyperactivity of childhood onset. N. Engl. J. Med..

[77] Vilsbøll T, Knop FK, Krarup T, Johansen A, Madsbad S, Larsen S (2003). The pathophysiology of diabetes involves a defective amplification of the late-phase insulin response to glucose by glucose-dependent insulinotropic polypeptide-regardless of etiology and phenotype. J. Clin. Endocrinol. Metab..

[78] Mosavat M, Omar SZ, Jamalpour S, Tan PC (2020). Serum glucose-dependent insulinotropic polypeptide (GIP) and glucagon-like peptide-1 (GLP-1) in association with the risk of gestational diabetes: A prospective case-control study. J. Diabetes Res..

[79] Ji J, Chen T, Sundquist J, Sundquist K (2018). Type 1 diabetes in parents and risk of attention deficit/hyperactivity disorder in offspring: A population-based study in Sweden. Diabetes Care.

[80] Xiang AH, Wang X, Martinez MP, Getahun D, Page KA, Buchanan TA (2018). Maternal gestational diabetes mellitus, type 1 diabetes, and type 2 diabetes during pregnancy and risk of ADHD in offspring. Diabetes Care.

[81] Xu G, Liu B, Yang W, Snetselaar LG, Jing J (2021). Association of attention-deficit/hyperactivity disorder with diabetes mellitus in US adults. J. Diabetes..

[82] Protic D, Aydin EY, Tassone F, Tan MM, Hagerman RJ, Schneider A (2019). Cognitive and behavioral improvement in adults with fragile X syndrome treated with metformin-two cases. Mol. Genet. Genomic Med..

[83] Faivre E, Gault VA, Thorens B, Hölscher C (2011). Glucose-dependent insulinotropic polypeptide receptor knockout mice are impaired in learning, synaptic plasticity, and neurogenesis. J. Neurophysiol..

[84] Faivre E, Hamilton A, Hölscher C (2012). Effects of acute and chronic administration of GIP analogues on cognition, synaptic plasticity and neurogenesis in mice. Eur. J. Pharmacol..

[85] Hölscher C (2020). Brain insulin resistance: Role in neurodegenerative disease and potential for targeting. Expert Opin. Investig. Drugs..

[86] Verma MK, Goel R, Krishnadas N, Nemmani KVS (2018). Targeting glucose-dependent insulinotropic polypeptide receptor for neurodegenerative disorders. Expert Opin. Ther. Targets..

[87] Sherry ST, Ward MH, Kholodov M, Baker J, Phan L, Smigielski EM (2001). dbSNP: The NCBI database of genetic variation. Nucleic Acids Res..

[88] Piñero J, Bravo À, Queralt-Rosinach N, Gutiérrez-Sacristán A, Deu-Pons J, Centeno E (2017). DisGeNET: A comprehensive platform integrating information on human disease-associated genes and variants. Nucleic Acids Res..

[89] Pejaver V, Mooney SD, Radivojac P (2017). Missense variant pathogenicity predictors generalize well across a range of function-specific prediction challenges. Hum. Mutat..

[90] Mi H, Ebert D, Muruganujan A, Mills C, Albou LP, Mushayamaha T (2021). PANTHER version 16: A revised family classification, tree-based classification tool, enhancer regions and extensive API. Nucleic Acids Res..

[91] Tang H, Thomas PD (2016). PANTHER-PSEP: Predicting disease-causing genetic variants using position-specific evolutionary preservation. Bioinformatics.

[92] Capriotti E, Calabrese R, Casadio R (2006). Predicting the insurgence of human genetic diseases associated to single point protein mutations with support vector machines and evolutionary information. Bioinformatics.

[93] Adzhubei IA, Schmidt S, Peshkin L, Ramensky VE, Gerasimova A, Bork P (2010). A method and server for predicting damaging missense mutations. Nat. Methods..

[94] Choi Y, Chan AP (2015). PROVEAN web server: A tool to predict the functional effect of amino acid substitutions and indels. Bioinformatics.

[95] Sim NL, Kumar P, Hu J, Henikoff S, Schneider G, Ng PC (2012). SIFT web server: Predicting effects of amino acid substitutions on proteins. Nucleic Acids Res..

[96] Cheng J, Randall A, Baldi P (2006). Prediction of protein stability changes for single-site mutations using support vector machines. Proteins.

[97] Wang Z, Huang C, Lv H, Zhang M, Li X (2020). In silico analysis and high-risk pathogenic phenotype predictions of non-synonymous single nucleotide polymorphisms in human Crystallin beta A4 gene associated with congenital cataract. PLoS ONE.

[98] Wang YF, Ding J, Wang F, Bu DF (2004). Effect of glycine substitutions on alpha5(IV) chain structure and structure-phenotype correlations in Alport syndrome. Biochem. Biophys. Res. Commun..

[99] Ashkenazy H, Abadi S, Martz E, Chay O, Mayrose I, Pupko T (2016). ConSurf 2016: An improved methodology to estimate and visualize evolutionary conservation in macromolecules. Nucleic Acids Res..

[100] Venselaar H, Te Beek TA, Kuipers RK, Hekkelman ML, Vriend G (2010). Protein structure analysis of mutations causing inheritable diseases. An e-Science approach with life scientist friendly interfaces. BMC Bioinform..

[101] Letunic I, Bork P (2018). 20 years of the SMART protein domain annotation resource. Nucleic Acids Res..

[102] Potter SC, Luciani A, Eddy SR, Park Y, Lopez R, Finn RD (2018). HMMER web server: 2018 update. Nucleic Acids Res..

[103] van Zundert GCP, Rodrigues JPGL, Trellet M, Schmitz C, Kastritis PL, Karaca E (2016). The HADDOCK2.2 web server: User-friendly integrative modeling of biomolecular complexes. J. Mol. Biol..

[104] Jiménez-García B, Pons C, Fernández-Recio J (2013). pyDockWEB: A web server for rigid-body protein-protein docking using electrostatics and desolvation scoring. Bioinformatics.

[105] Cheng TM, Blundell TL, Fernandez-Recio J (2007). pyDock: Electrostatics and desolvation for effective scoring of rigid-body protein-protein docking. Proteins.

[106] Yan Y, Tao H, He J, Huang SY (2020). The HDOCK server for integrated protein-protein docking. Nat. Protoc..

[107] Alekseenko A, Ignatov M, Jones G, Sabitova M, Kozakov D (2020). Protein-protein and protein-peptide docking with ClusPro server. Methods Mol Biol..

[108] Ramírez-Aportela E, López-Blanco JR, Chacón P (2016). FRODOCK 2.0: Fast protein-protein docking server. Bioinformatics.

[109] Ghoorah AW, Devignes MD, Smaïl-Tabbone M, Ritchie DW (2016). Classification and exploration of 3D protein domain interactions using Kbdock. Methods Mol. Biol..

[110] Ward LD, Kellis M (2016). HaploReg v4: Systematic mining of putative causal variants, cell types, regulators and target genes for human complex traits and disease. Nucleic Acids Res..

