# Supplementary material for: ADGRL3 genomic variation implicated in neurogenesis and ADHD links functional effects to the incretin polypeptide GIP
Source: Sci Rep. 2022 Sep 23;12:15922. doi: 10.1038/s41598-022-20343-z (PMC9508192; doi:10.1038/s41598-022-20343-z)
Supplement: Supplementary file 1 — Supplementary Information 1. [file 41598_2022_20343_MOESM1_ESM.docx]

Supplementary Material for:

*ADGRL3* Genomic Variation Implicated in Neurogenesis and ADHD Links Functional Effects to the Incretin Polypeptide GIP

Oscar M. Vidal^1,#,*^, Jorge I. Vélez^1,*^, Mauricio Arcos-Burgos^2,*,#^

July 15, 2022

^1^ *Universidad del Norte, Barranquilla, Colombia 081007*.

^2^ *Grupo de Investigación en Psiquiatría (GIPSI), Departamento de Psiquiatría, Instituto de Investigaciones Médicas, Facultad de Medicina, Universidad de Antioquia, Medellín, Colombia* *050010.*

^#^ *These authors contributed equally to this work.*

*^*^* Correspondence to be directed to

Dr. Oscar M. Vidal, PhD Dr. Jorge I. Vélez, PhD

Assistant Professor, Assistant Professor,

Department of Medicine, Department of Industrial Engineering,

Universidad del Norte, Universidad del Norte,

Km 5 vía Puerto Colombia, Km 5 vía Puerto Colombia,

Building F, Room 2-4F, Building K, Room 7-3K,

Barranquilla, Colombia. Barranquilla, Colombia.

E-mail: [oorjuela@uninorte.edu.co](mailto:oorjuela@uninorte.edu.co) E-mail: [jvelezv@uninorte.edu.co](mailto:jvelezv@uninorte.edu.co)

Phone +57 (605) 3509509 ext. 3954 Phone +57 (605) 3509509 ext. 3279

The authors have declared they have no financial interests to disclose.

**ORCIDs**

OMV: <https://orcid.org/0000-0002-7807-1242>

JIV: <https://orcid.org/0000-0002-3146-7899>

MAB: <https://orcid.org/0000-0002-8529-0574>

**Figure 1S.** Prediction of ADGRL3 using AlphaFold Protein Structure Database. Confidence bands are used to color-code the residues in the 3D viewer. Regions <50 pLDDT may be unstructured in isolation.


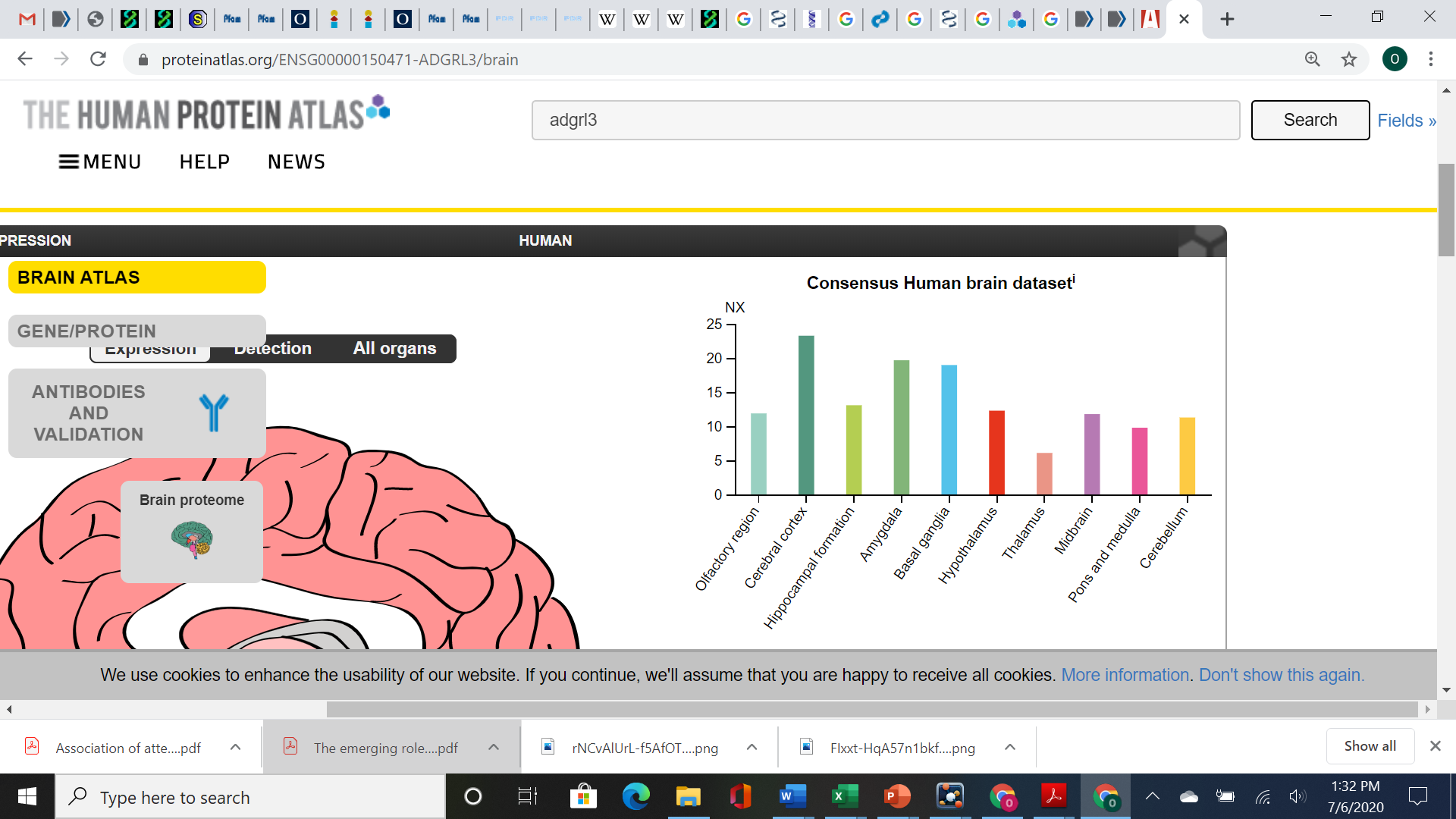


**Figure 2S.** ADGRL3 human mRNA expression levels in key brain areas. These areas modulate high-level brain-controlled functions, including attention, executive function and organization. Source: <https://www.proteinatlas.org/ENSG00000150471-ADGRL3/brain>).

**Figure 3S.** AlphaFold2 prediction analysis of the 3D protein structure for **(a)** WT ADGRL3 HormR domain and **(b)** the ADGRL3 HormR domain when the R465Q aminoacid change is introduced (cyan arrow). White double-head arrows point highlight changes in 3D protein structure.

**Table 2S.** Results of scoring functions for protein-protein docking using different *in-silico* approaches. RMDS: Root Mean Square Deviation in Å.

| **Docking program** | **Van der Waals energy** | **Electrostatic energy** | **Desolvation**  **energy** | **Rank** | **Score/ *Z*-score** |
| --- | --- | --- | --- | --- | --- |
| HADDOK | -74.2 +/- 5.0 | -282.1+/- 34.1 | -25.8+/- 1.2 | 1 | -1.9 |
| PyDock | 44.85 | -12.584 | -11,629 | 1 | -94.728 |
| HDOCK | - | - | - | 10 | -191.67 |
| ClusPro | Center | - | - | 2 | -824.5 |
| FRODOCK | | - | - | 1 | 2637.23 |
| KBDOCK |  | - | - | 2 | RMSD=1.31 |
